# Paternal obesity induces changes in sperm chromatin accessibility and has a mild effect on offspring metabolic health

**DOI:** 10.1016/j.heliyon.2024.e34043

**Published:** 2024-07-05

**Authors:** Iasim Tahiri, Sergio R. Llana, Júlia Fos-Domènech, Maria Milà-Guash, Miriam Toledo, Roberta Haddad-Tóvolli, Marc Claret, Arnaud Obri

**Affiliations:** a Neuronal Control of Metabolism Laboratory, Institut d'Investigacions Biomèdiques August Pi i Sunyer, Barcelona, Spain; b CIBER de Diabetes y Enfermedades Metabólicas Asociadas (CIBERDEM), Barcelona, Spain; c School of Medicine, Universitat de Barcelona, Barcelona, Spain

**Keywords:** Paternal obesity, Sperm epigenetic, Metabolic health, Epigenetic inheritance

## Abstract

The increasing global burden of metabolic disorders including obesity and diabetes necessitates a comprehensive understanding of their etiology, which not only encompasses genetic and environmental factors but also parental influence. Recent evidence has unveiled paternal obesity as a contributing factor to offspring's metabolic health via sperm epigenetic modifications. In this study, we investigated the impact of a Western diet-induced obesity in C57BL/6 male mice on sperm chromatin accessibility and the subsequent metabolic health of their progeny. Utilizing Assay for Transposase-Accessible Chromatin with sequencing, we discovered 450 regions with differential accessibility in sperm from obese fathers, implicating key developmental and metabolic pathways. Contrary to expectations, these epigenetic alterations in sperm were not predictive of long-term metabolic disorders in offspring, who exhibited only mild transient metabolic changes early in life. Both male and female F1 progeny showed no enduring predisposition to obesity or diabetes. These results underscore the biological resilience of offspring to paternal epigenetic inheritance, suggesting a complex interplay between inherited epigenetic modifications and the offspring's own developmental compensatory mechanisms. This study calls for further research into the biological processes that confer this resilience, which could inform interventional strategies to combat the heritability of metabolic diseases.

## 1 Introduction

The rising prevalence of metabolic conditions like obesity, diabetes, and cardiovascular disease highlights a major global public health concern [1,2]. Those diseases arise from an intricate interaction of genetic, environmental, and behavioral variables [[3], [4], [5]]. Recent data stresses the crucial importance of parental contributions to the metabolic wellness of offspring, indicating that the health of parents at the time of conception can have significant impact on the future health trajectory of the following generation [6]. Maternal influences, such as obesity and diabetes during pregnancy, predispose offspring to similar metabolic disorders through mechanisms including altered fetal programming and changes in the uterine environment [7,8]. These maternal contributions are now complemented by emerging evidence on the significant role of paternal factors. Epidemiological studies have established a correlation between the metabolic health of children and parameters such as the paternal body weight, dietary habits, and overall lifestyle [[9], [10], [11], [12], [13], [14]]. Animal studies have emerged as a crucial method for understanding the mechanisms that explain how paternal metabolism affects offspring health. This is especially important because human epidemiological research has inherent limitations. The initial data came from a study that showed that the offspring of fathers who were fed a high-fat diet (HFD) had metabolic alterations such as decreased insulin secretion and glucose intolerance [15]. Later, it was shown that paternal obesity can also lead to alteration that persist in the second generation (F2) [16]. Recent evidence suggests that long-term exposure across multiple generations to obesogenic diet can create an epigenetic susceptibility to metabolic diseases [17]. This further emphasizes the persistent effect of a father's eating habits on the health of future generations. In fruit flies, short-term increases in dietary sugar intake in fathers resulted in obesity, higher triglyceride levels, and increased food intake in the first generation (F1) progeny [18].

Epigenetic modifications involve various processes that can influence gene expression, such as DNA methylation, histone modifications, and noncoding RNAs. The mentioned modifications possess the capacity to influence gene expression while keeping the underlying DNA sequence intact [19]. Noncoding RNA families seem to have a predominant role in transferring the father's influence to offspring metabolic health [20]. Initial findings suggested that offspring generated through in vitro fertilization using sperm from males who were obese and carried small RNAs showed early indications of poor glucose tolerance and insulin resistance. These indicators were observed as early as seven weeks of life [21]. Small RNAs are transmitted in a dynamic manner from the epididymis to mature sperm [22,23]. Among these, transfer RNA-derived short RNAs (tsRNAs) have a role in controlling gene silence, responding to stress, and regulating cellular metabolism [24]. Specifically, tsRNA, generated by the enzyme DNA methyltransferase 2 (DNMT2), are essential and sufficient to transmit paternal acquired metabolic disorders to the offspring [25]. Sperm micro-RNA (miRNA) profiles are greatly affected by diet and exercise, and it has been suggested to have repercussions in glucose metabolism in adult offspring [[26], [27], [28]]. Research conducted on rats has demonstrated that there is a similarity in the sperm DNA methylation pattern of obese fathers and their offspring [16]. Similarly, a low protein diet affects sperm DNA methylation in mice, which can be observed in later generations [29]. Sperm chromatin structure is unique, characterized by a predominantly protamine-based composition that replaces most histones during the process of spermatogenesis. Our current understanding of sperm chromatin structure is notably incomplete, and this lack of comprehensive knowledge challenges our grasp of its true complexity and implications for epigenetic inheritance. Initial research has detected the existence of significant epigenetic modifications in sperm. Initial research has discovered the existence of significant epigenetic markings in sperm. One such example is the observation of histone H3 lysine 27 trimethylation (H3K27me3) on genes that are prepared for activation in the early stages of embryonic development [30,31]. Males who consume a high-fat diet have altered H3 content at critical genes related to development [32].

Despite notable progress in gaining insights into the impact of epigenetic changes in sperm in the transmission of metabolic traits the exploration of sperm chromatin accessibility in this area has not been thoroughly investigated. Some reports have examined it in different contexts but not specifically in relation to the inheritance of metabolic traits [33]. We aimed to better understand the effects of sperm chromatin accessibility from the father on the metabolic health of the offspring. We utilized Assay for Transposase-Accessible Chromatin with sequencing (ATAC-seq), an unbiased approach to map chromatin accessible sites genome-wide. By feeding male mice an obesogenic diet rich in fat and carbohydrates (a so-called Western diet or WD) to induce obesity, we were able to investigate the subsequent changes in sperm chromatin accessibility. Our findings reveal that exposure to a WD significantly alters the accessibility of numerous genes implicated in a variety of biological pathways, particularly those related to metabolism and development. Nonetheless, mating these obese males with lean females resulted in offspring that exhibited only mild metabolic changes in body weight and glucose homeostasis with and no apparent predisposition to obesity or diabetes in the F1 generation. This indicates that while diet induces alterations in sperm chromatin accessibility that may influence progeny, the resultant metabolic effects are not pronounced.

## 2 Material and methods

### 2.1 Animal care and diets

The University of Barcelona Ethics Committee approved all animal research (Approval No. CEEA-168/20), which was conducted in accordance with the current laws in Catalonia, Spain, and Europe. Three-week-old C57BL/6JRccHsd virgin male and female mice were acquired from Envigo. At four weeks old, male mice were randomly assigned to two groups: the first group received a control diet (#2014S, maintenance diet with 14 % protein; Envigo), while the second group was provided with a Western diet (#D12079B, 40 % kcal from fat and 43 % kcal from carbohydrates; Research Diets) for a duration of six months. Female mice were kept in the facility until they reached reproductive maturity. F1 offspring were kept on the control diet from birth until day 21 (P21). Mice were housed in a controlled environment, maintaining a temperature range of 20-24 °C and a 12-h light-dark cycle. The mice's health condition was constantly evaluated.

#### 2.1.1 Breeding strategy

At 28 weeks of age, male mice from both the control and Western diet groups were paired with nulliparous 8-week-old females for one week. During this period, the females were provided with the same diet as their respective male partners. Although exposing females to a Western diet during the mating period (up to one week) may have some impact on oocytes and subsequently on offspring development. After mating, the females were separated and housed with another female from the same paternal diet group and were switched back to the control diet until the offspring were born. Starting from day 18 of pregnancy, the females were observed daily. Litter size equalization was implemented to ensure that each mother reared an equal number of offspring, specifically seven to eight pups per female. When adjusting litter sizes, we removed the lightest pup whenever possible. If this was not feasible, the adjustments were done randomly. We did not take the sex of the pups into account during litter size adjustment. This approach was used to minimize variation between litters caused by competition for postpartum nutrients.

Non-pregnant females or those that had litter loss were excluded from the study. At P21, the first-generation (F1) offspring were separated from their mothers and organized into groups. F1 offspring from each paternal group were divided into two subgroups: one subgroup remained on the control diet, while the other subgroup received a Western diet starting at 12 weeks of age. We ensured that each litter was represented in both subgroups.

### 2.2 Mouse mature sperm extraction

Mouse mature sperm extraction was carried out in accordance with the protocol used for murine in vitro fertilization [34]. For each dissection, a Petri dish was prepared containing 1 mL of 1x PBS at its center, subsequently overlaid with paraffin oil (# 76235, Sigma). Following euthanasia of the male mouse by cervical dislocation, both caudae epididymides were excised and positioned on the paraffin oil in the Petri dish. Each cauda was incised midway using scissors, releasing the sperm clusters, which were then coaxed towards the 1x PBS at the dish's center. Following a 10-min period of incubation at ambient temperature, the phosphate-buffered saline (PBS) solution containing the sperm was gathered and centrifugated at 600g at a temperature of 4 °C for a duration of 5 min. The sperm was thereafter washed twice with 1x PBS.

### 2.3 Gene expression analysis by quantitative PCR

Sperm total RNA was isolated from mature sperm using TRIZOL (#15596026, Invitrogen) using a standard protocol. The conversion of total RNAs into cDNA was performed using the High-Capacity cDNA Reverse Transcription Kit (#4368813, Thermo Fisher). Quantitative PCR (qPCR) was conducted using an ABI Prism 7900 HT system (Applied Biosystems). The qPCR analysis utilized Taqman Gene Expression assay probes (Applied Biosystems) for the following genes: *Prmt1* (Mm01342731_g1, Thermo Fisher), *Prmt2* (Mm03048199_m1, Thermo Fisher), *C-Kit* (Mm00445212_m1, Thermo Fisher) and *E-Cadherin* (Mm01247357_m1, Thermo Fisher). Gene expression was normalized using the relative levels of *Prmt1* as a reference.

### 2.4 Biomarkers quantification assays

Plasma insulin levels were quantified in F1 offspring using enzyme-linked immunosorbent assay (ELISA) kit (Crystal Chem #90080) and by following the protocol provided by the manufacturer. Hepatic triglycerides were quantified on liver extract using a commercial kit and following manufacturer instructions (BioVision #K622-100).

### 2.5 Metabolic function assays

All metabolic assays, including GTT, ITT, and PTT/GlyTT, were conducted in the F1 offspring. These tests were performed at postnatal day 21 (P21), 12 weeks of age, and 24 weeks of age (at the end of the 12-week Western diet feeding).

#### 2.5.1 Body weight and blood collection

Body weights were measured using a precision scale. Blood samples were collected from the offspring via tail vein using a capillary collection system with EDTA (NC9141704, Sarstedt). The blood samples were then centrifuged at 600g for 10 min at 4 °C and stored at −80 °C until further analysis.

#### 2.5.2 Glucose tolerance test (GTT)

GTT was conducted by administering a single dose of glucose (2 g/kg of body weight – 620724, Fresenius Kabi) through an intraperitoneal injection in mice that had fasted for 6 h. Glucose was dissolved in sterile saline solution. Animals were fasted for 6 h. Blood samples were obtained from the tail before to glucose administration (0min) and at 15, 30, 60, and 120min thereafter. The blood glucose concentration was promptly assessed using a glucose meter (Nova Pro Biomedical).

#### 2.5.3 Insulin tolerance test (ITT)

ITT was performed by administering a single injection of insulin (0.5 IU/kg of body weight - #C.N 710008.9, Lilly). Animals were fasted for 6 h. Glucose levels in the blood were assessed before the injection (0 min) and at 15, 30, 60, and 120 min after the injection. If the blood glucose level of a mouse dropped below 30 mg/dL, it was administered a rescue intraperitoneal injection of glucose, and thereafter removed from the experiment. Mouse tail vein blood samples were collected, and blood glucose concentrations were promptly assessed using a glucose meter (Nova Pro Biomedical).

#### 2.5.4 Pyruvate and glycerol tolerance test (PTT/GlyTT)

PTT and GlyTT was carried out by administering one intraperitoneal dose of sodium pyruvate or glycerol at a concentration of 1 g/kg. The sodium pyruvate (P3662, Sigma) or glycerol (G551, Sigma) was dissolved in a saline solution. Animals were fasted for 16 h. Pre-injection blood glucose concentrations were assessed at time 0 min, and post-injection measurements were taken at 15, 30, 60, and 120 min. Mouse tail vein blood samples were obtained, and blood glucose concentrations were promptly assessed using a glucose meter (Nova Pro Biomedical).

### 2.6 Assay for transposase-accessible chromatin followed by sequencing

Mature sperm from six F0 CD males and four F0 WD males were extracted. ATAC-seq was performed on individual samples and according to a modified Omni-ATAC protocol [35]. The mature sperm was counted using a Neubauer chamber. Nuclei from 100,000 mature sperm were isolated with a lysis solution at cold temperature, composed of 10 mM Tris-HCl (pH 7.5), 10 mM NaCl, 3 mM MgCl2, 0.1 % IGEPAL, 0.1 % Tween-20, and 0.01 % Digitonin. These nuclei were then treated with a transposase mixture that included 1 % digitonin and 10 % Tween-20, and incubated at 37 °C for 30 min. After tagmentation, proteinase K and 150uM of dithiothreitol (DTT) were added and allowed to incubate for a duration of 2 h at 55 °C. The tagmentated genomic DNA was retrieved using Phenol:Chlorophorm:Isoamyl alcohol and then precipitated with ethanol. Library amplification was achieved using 2X KAPA HiFi mix and 25 μM indexed primers under PCR conditions that included an initial denaturation at 98 °C for 30 s, 11 cycles of denaturation at 98 °C for 10 s, annealing at 63 °C for 30 s, and extension at 72 °C for 1 min. Each ATAC-seq experiment was conducted with 4 and 6 biological replicates on control diet (F0 CD) and Western diet (F0 WD) groups respectively. The libraries were processed using an Illumina NextSeq2000 sequencing platform, operating in paired-end mode with a read length of 2 × 50 bp. Each sample generated a minimum of 60 million paired-end reads after completing all quality control procedures.

### 2.7 ATAC-seq processing and analysis

TrimGalore v0.6.10 was utilized for adapter and quality trimming. Afterward, we aligned the paired-end sequences to mouse reference genome (mm10) employing Bowtie2 v2.4.5, with settings –N 1 -L 25 -X 2000 -k 20, while avoiding mixed and discordant alignments [36]. Duplicate reads were identified and removed with Picard v2.24.0. Filtering of unmapped sequences, low-quality alignments (q < 5), and duplicates was carried out using Samtools [37]. We also excluded sequences aligning to mitochondrial DNA, chromosome Y, and alternative chromosomes. Aligned reads were shifted +4/-5 bp for forward/reverse strands respectively, accounting for the 9-bp duplication created by DNA repair of the nick by transposase, achieving base-pair resolution of transcription factor motif-related analyses. We only analyzed fragments between 50 and 115 bp). We performed peakcalling using MACS2 v2.2.7.1 with parameters -q 0.05 -f BED --nomodel --shift −100 --extsize 200 --keep-dup all --call-summits; using BED files (converted from BAM) as input [38]. A consensus peak matrix was created merging the narrowPeak output files from MACS2, this was used to create a matrix of peak counts per sample to keep peaks detected in at least two samples. Differential accessibility was analyzed using DESeq2 v1.40.2, comparing consensus peaks between control and Western diet groups [39], with accessibility differences defined by log2 fold change thresholds of >1 for increased, and < −1 for decreased, accessibility under a Western diet, considering a p-value <0.05. Nearest transcription start sites to differentially accessible peaks were identified using the ChIPseeker's annotatePeak function v1.36 and TxDb.Mmusculus.UCSC.mm10.knownGene v3.10 as reference [40]. Gene Ontology enrichment was performed with gprofiler2, adhering to an FDR <0.05 [41]. Transcription factor motif enrichment analyses were performed using HOMER v4.11.1 findMotifsGenome.pl with mm10 (v6.4) genome and -size given parameter; identifying known transcription factor motif sequences [42]. Western diet peaks with increased differential accessibility were compared to the ones of control diet and vice versa. R code scripts are available at https://github.com/SergioRodLla/Paternal-obesity-induced-changes-in-sperm-chromatin-accessibility-and-have-mild-effect-on-offspring.

### 2.8 Statistical analysis

Data is presented as the mean value accompanied by their respective standard errors (SEM). Statistical evaluations were conducted with GraphPad Prism software (version 9.0). Details on the specific statistical tests applied to each study are indicated in the legends accompanying the figures.

## 3 Results and discussion

### 3.1 Obesity modifies sperm chromatin accessibility

In order to examine the possible influence of alterations in sperm chromatin accessibility on the transfer of obesity susceptibility to offspring, we initially produced obese male mice through the use of a diet-induced obesity protocol. Male mice that were four weeks old were provided with either a control diet (referred to as F0 CD) or a Western diet (referred to as F0 WD) for a duration of six months. Most studies use high-fat-diet (sometimes ≥60 % fat) that most likely results in deficiencies of important nutrients and fiber, possibly contributing to the observed deleterious effects. Here we use a standardized rodent diet that better mimics a human Western diet [43]. As anticipated, male mice consuming a WD gained significantly more weight and developed diabetes when compared to male fed a CD (Supplementary Figs. 1A–D), which validated our obesity model.

To check whether sperm chromatin accessibility is affected by obesity, we opted for the ATAC-seq. First, we isolated sperm from the cauda epididymis of male mice from either F0 CD or F0 WD groups. A qPCR was conducted to analyze the quality control of the sperm preparation. This analysis confirmed a high enrichment of the mature sperm marker *Prmt2*, with no detectable contamination from testicular germ cells (as indicated by the absence of *C-kit*) or epithelial cells (lack of *E-cadherin*) (Supplementary Fig. 2A). We also compared our control ATAC-seq dataset to previously published sperm ATAC-seq datasets [44] and found a significant match confirming the validity of our methodology (Supplementary Fig. 2B).

**Fig. 1:**
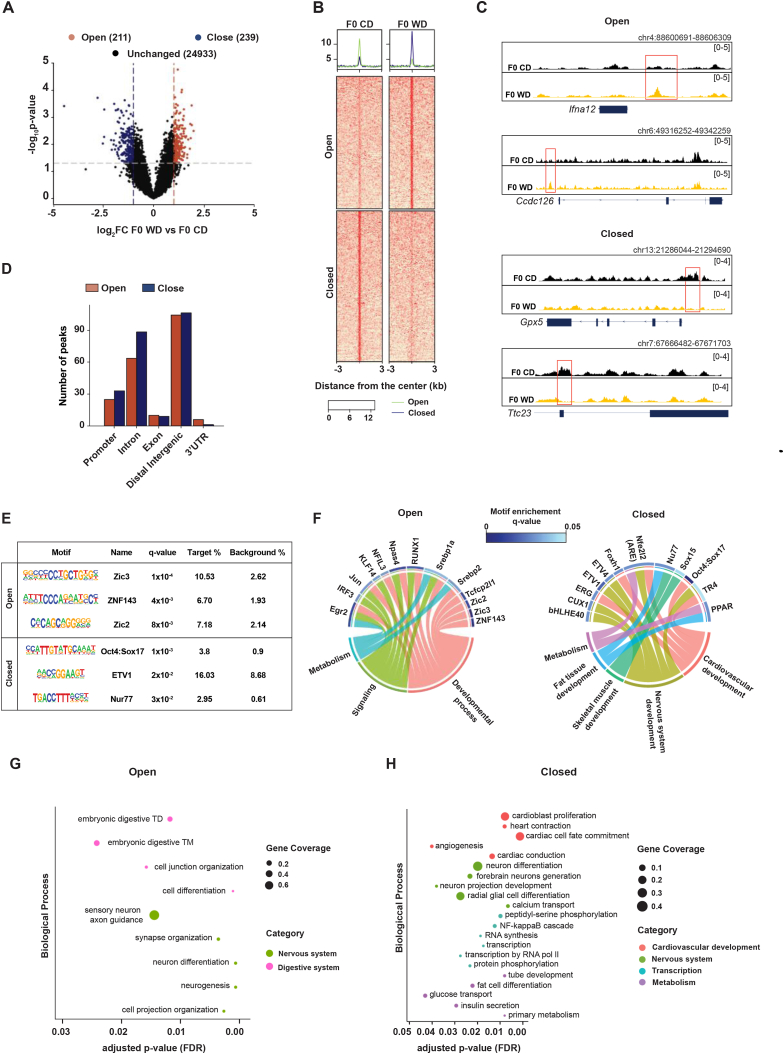
Obesity induces change in sperm chromatin accessibility. (A) Volcano plot showing differential sperm chromatin accessibility in F0 males on Western diet (F0 WD, n = 4) or control diet (F0 CD, n = 6), with a specified fold change (FC) cutoff of log_2_(FC) > 1 (red) and log_2_(FC) < −1 (blue) respectively, and a significance cutoff of P-value <0.05 [-log_10_(P-value) > 1.3]. Each point represents an ATAC-seq peak. (B) Heatmap depicting peak intensities with a color gradient, highlighting differences in sperm chromatin accessibility between diets. (C) Genome browser tracks showing increased (open) and decreased (closed) accessibility regions at different genomic loci. Tracks display averaged signals for both F0 CD (black, n = 6) and F0 WD (yellow, n = 4) samples. (D) Distribution of annotation categories for differentially closed (blue) and open (red) chromatin regions in sperm from F0 CD vs F0 WD. UTR, untranslated regions. (E) Motif enrichment analysis conducted with HOMER for open and closed chromatin regions, indicating the top-scoring motifs, q-values, best-match transcription factors, and percentage of target versus background. Top 3 candidates are shown for open and closed regions. (F) Chord diagrams representing biological process pathways related to enriched TF motifs for both open (left) and closed (right) chromatin regions. Blue shading of grids adjacent to TF motifs indicates q-value (Benjamini corrected p-value). (G–H) Gene Ontology (GO) analyses for biological processes associated with open (G) and closed (H) chromatin regions. Fisher's one-tailed test (cumulative hypergeometric probability) P-values were adjusted using false discovery rate (FDR). TM: tract morphogenesis, TD: tract development. (For interpretation of the references to color in this figure legend, the reader is referred to the Web version of this article.)

The subsequent ATAC-seq analysis revealed 211 differentially open regions (p-value <0.05, log2 fold change >1) and 239 differentially closed regions (p-value <0.05, log2 fold change < −1) when comparing sperm from the F0 WD group to the F0 CD group (Fig. 1A–B, Supplementary Figs. 3 and 4). Representative gene tracks are shown as examples of open and closed regions (Fig. 1C).

Further annotation analysis suggested that both open and closed regions were predominantly located within distal intergenic areas (50 % and 44.77 %, respectively) and introns (30.48 % and 37.24 %, respectively), implying that obesity might influence chromatin accessibility through enhancers at a distal level (Fig. 1D). This could suggest a nuanced regulatory network in which obesity does not directly alter gene expression but rather modulates the potential for expression in response to subsequent environmental cues. Additionally, we compared the open and closed regions in sperm (Fig. 1B) to a previous study that mapped H3K4me3 in the sperm of obese mice [45]. We found that only a small number of our regions matched with H3K4me3 enrichment (Supplementary Fig. 2C). This limited overlap is due to our regions with differential accessibility being primarily in distal intergenic and intron areas of the genome, whereas H3K4me3 is predominantly found in promoter regions.

Further motif enrichment analysis on the open and closed chromatin regions identified motifs associated with 13 transcription factors for open regions (q-value <0.05) and with 12 transcription factors for closed regions (q-value <0.05) (Fig. 1E and Supplementary Figs. 5A–B). Notably, these transcription factors played a role in the regulation of developmental process, metabolism and signaling pathways (Fig. 1F). In addition, a gene ontology pathway analysis of the differentially accessible regions reinforced the notion that nervous system, transcription, and metabolism could be influenced by a Western diet in sperm (Fig. 1G and H).

The implication of these findings is particularly significant when considered alongside the results of similar studies. Notably, in humans, sperm DNA methylation patterns were altered in obese males and related with comparable pathways that we uncovered [46].

We therefore conclude that obesity potentially induces epigenetic modifications in mature sperm, as indicated by alterations in sperm chromatin accessibility which could regulate the availability to specific transcription factors governing distinct biological processes.

### 3.2 Paternal obesity-induced changes in sperm chromatin have a mild effect on the metabolic health of the first-generation offspring

**Fig. 2:**
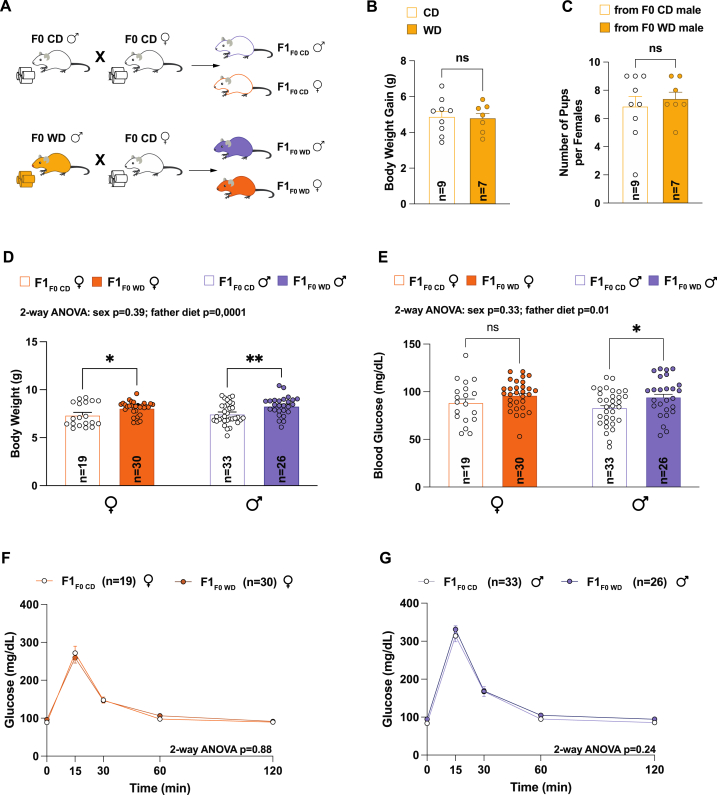
Changes in the father's sperm chromatin accessibility can slightly affect the metabolic health of their offspring in early life. (A) Breeding strategy used in this study to generate the F1 generation: Obese males (F0 WD) or lean males (F0 CD) were mated with virgin lean females for one week, producing offspring from obese fathers (F1_F0__WD_) and lean fathers (F1_F0 CD_). (B) Body weight gain in females mated with males on either a control diet (CD, n = 9) or a Western diet (WD, n = 7) was assessed before and after the gestation period (C) Litter size per female mated with males on either a control diet (CD, n = 9) or a Western diet (WD, n = 7) (D) Body weight of F1 offspring at postnatal day 21 (P21) of females from both father types (F1_F0 CD_, n = 19; F1_F0__WD_, n = 30), and of males (F1_F0 CD_, n = 33; F1_F0__WD_, n = 26). Statistical significance was assessed using a two-way ANOVA test, followed by multiple comparisons using a Sidak test. (E) Blood glucose level after 6 h of fasting at P21 of females from both father types (F1_F0 CD_, n = 19; F1_F0__WD_, n = 30), and of males (F1_F0 CD_, n = 33; F1_F0__WD_, n = 26). Statistical significance was assessed using a two-way ANOVA test, followed by multiple comparisons using a Sidak test. (F–G) Glucose tolerance test at P21: (F) females from both father types (F1_F0 CD_, n = 19; F1_F0__WD_, n = 30), (G) males (F1_F0 CD_, n = 33; F1_F0__WD_, n = 26). Statistical significance was assessed using two-way repeated measures ANOVA test. Results are given as mean ± SEM. *, P ≤ 0.05; **, P ≤ 0.01. ns = not significant.

To explore the role of paternal obesity on the physiology of offspring by studying changes in the accessibility of sperm chromatin, we utilized a natural mating method rather than in vitro fertilization. This involved pairing 28-week-old obese (F0 WD) or lean male (F0 CD) mice with 8-week-old virgin females (Fig. 2A).

We also monitored our male breeder's fertility and found that whereas 90 % (a total of nine litters) of the females who mated with F0 CD males successfully produced offspring, only 70 % (a total of seven litters) of the females who mated with F0 WD males had a litter. Nonetheless, we did not find any variations in the number of offspring born to each female, regardless of whether they mated with F0 CD or F0 WD males (Fig. 2C).

**Fig. 3:**
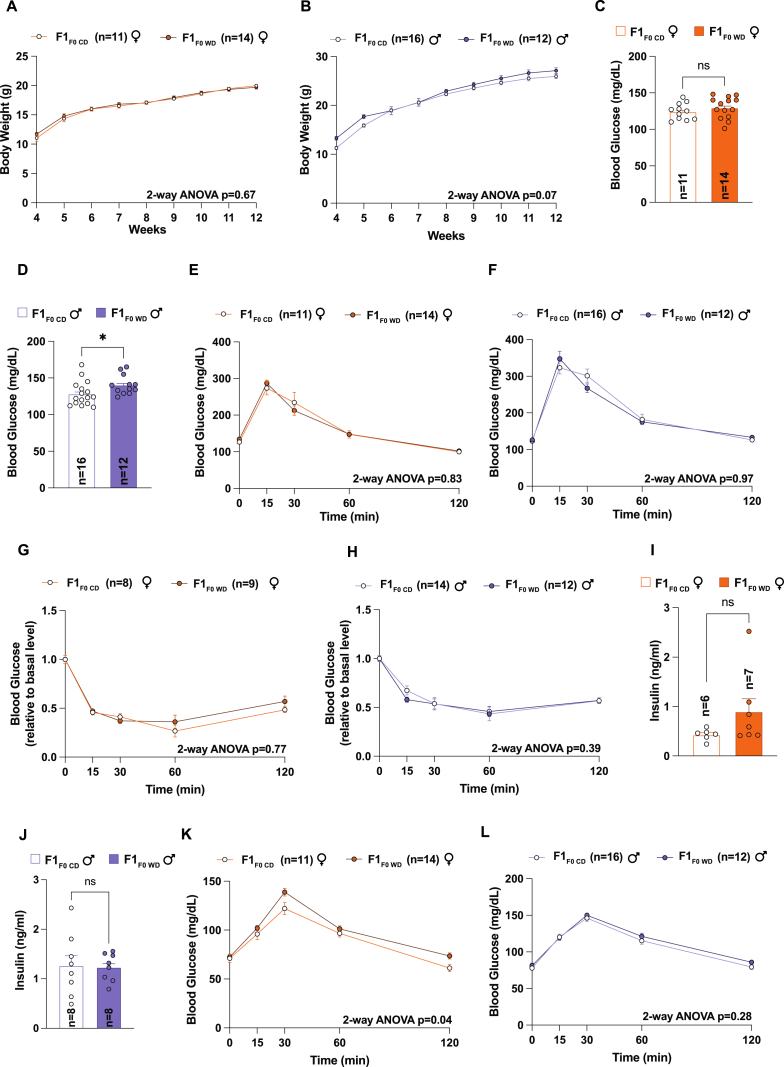
Alterations in a father's sperm chromatin accessibility led to inconsistent effects on the metabolic health of their adult offspring. (A–B) Body weight was monitored from 4 to 12 weeks of age for: (A) females from both father types (F1_F0 CD_, n = 11; F1_F0__WD_, n = 14), (B) males (F1_F0 CD_, n = 16; F1_F0__WD_, n = 12). Statistical significance was assessed using two-way repeated measures ANOVA test. (C–D) Blood glucose level after 6 h of fasting at 12 weeks of age: (C) females from both father types (F1_F0 CD_, n = 11; F1_F0__WD_, n = 14), (D) males (F1_F0 CD_, n = 16; F1_F0__WD_, n = 12). Statistical significance was assessed using Student's *t*-test. (E–F) Glucose tolerance test at 12 weeks of age: (E) females from both father types (F1_F0 CD_, n = 11; F1_F0__WD_, n = 14), (F) males (F1_F0 CD_, n = 16; F1_F0__WD_, n = 12). Statistical significance was assessed using two-way repeated measures ANOVA test. (G–H) Insulin tolerance test at 12 weeks of age: (G) females from both father types (F1_F0 CD_, n = 8; F1_F0__WD_, n = 9), (H) males (F1_F0 CD_, n = 14; F1_F0__WD_, n = 12). Statistical significance was assessed using two-way repeated measures ANOVA test. (I–J) Insulin level after 6 h of fasting at 12 weeks of age: (C) females from both father types (F1_F0 CD_, n = 6; F1_F0__WD_, n = 7), (D) males (F1_F0 CD_, n = 8; F1_F0__WD_, n = 8). Statistical significance was assessed using Student's *t*-test. (K–L) Pyruvate tolerance test at 12 weeks of age: (I) females from both father types (F1_F0 CD_, n = 11; F1_F0__WD_, n = 14), (J) males (F1_F0 CD_, n = 16; F1_F0__WD_, n = 12). Statistical significance was assessed using two-way repeated measures ANOVA test.

In the early life of the F1 progeny, we found offspring of both sexes from obese fathers (F1_F0__WD_) were heavier than those from lean fathers (F1_F0 CD_) (Fig. 2D). Male F1_F0__WD_ also presented elevated fasting glucose levels at P21, yet neither male nor female offspring showed glucose intolerance at weaning (Fig. 2E–G). Interestingly, the early metabolic changes observed did not persist into adulthood, as there were no noticeable differences in body weight between the two groups as they grew older (Fig. 3A and B). However, F1_F0__WD_ males continued to demonstrate higher fasting glucose levels in adult life (Fig. 3C–D), prompting further investigation into their glucose homeostasis through a series of tests, including glucose, insulin, and pyruvate tolerance tests (GTT, ITT and PTT) as well as fasting insulin concentration quantification. These assessments revealed no compromise in glucose homeostasis regulation among male F1_F0__WD_ offspring (Fig. 3F–H, L). In contrast, female offspring displayed a slight pyruvate intolerance, hinting at a subtle disruption in gluconeogenesis specifically related to pyruvate metabolism, while the glycerol tolerance test (GlyTT) did not indicate such disruption (Fig. 3E–G, K and Supplemental Fig. 6A). This suggests that other pathways of gluconeogenesis remain unaffected. Additionally, we measured hepatic triglyceride (TG) levels in these female offspring and found them to be unaltered (Supplemental Fig. 6B).

In summary, paternal obesity-related alterations in sperm chromatin appear to have a subtle impact on offspring's health. While initial increases in body weight were observed in both sexes, these differences normalized in adulthood. Glucose metabolism was also minimally affected, with variations observed between males and females. These nuanced results contrast with other reports demonstrating a more pronounced impact of paternal diet on offspring metabolism [15,47,48]. It is also important to acknowledge that the partial paternal transmission of metabolic traits observed in the offspring could be due to the specific time window chosen for the father's exposure to the Western diet.

Moreover, the differential impact observed between male and female offspring may indicate a sex-specific response to paternal epigenetic marks. This observation aligns with previous reports suggesting that the paternal environment can exert distinct effects on the metabolism of male versus female progeny [49,50]. Our study differs from prior published studies by simulating natural mating conditions [21,25,48]. Consequently, we also provide a valuable perspective on how the environment at the time of conception can amplify the contribution of paternal sperm epigenetics on next generation health.

### 3.3 Paternal obesity-induced changes in sperm chromatin do not predispose offspring to develop obesity or diabetes

**Fig. 4:**
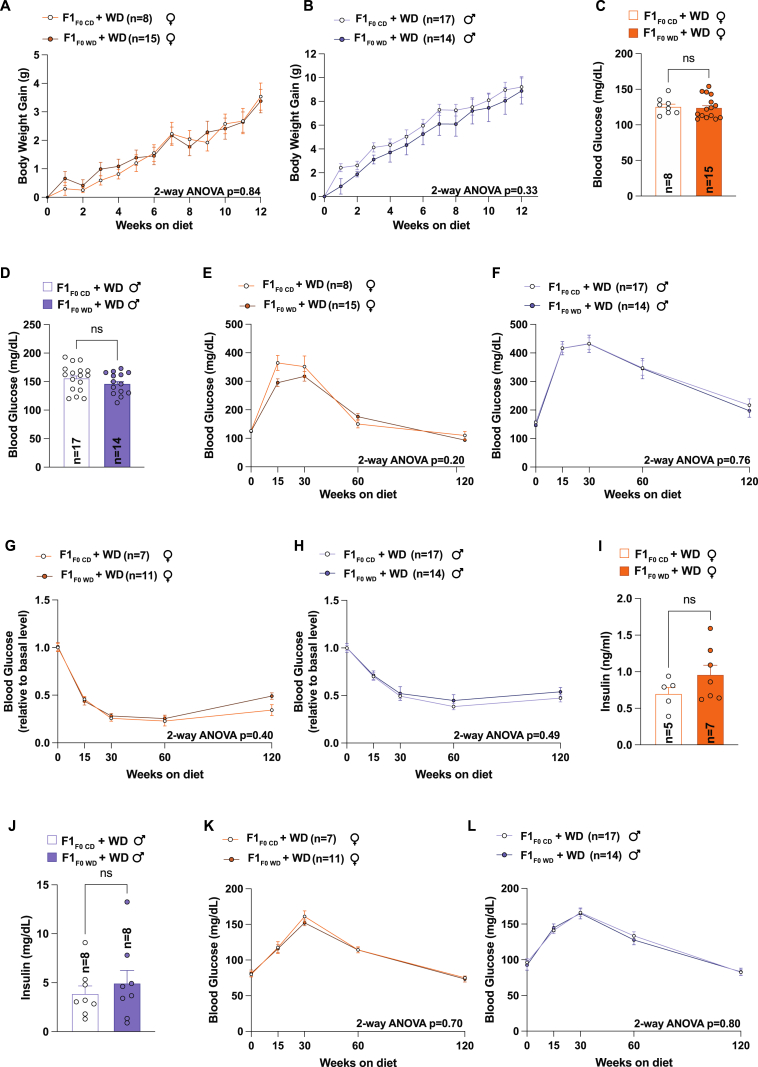
Change in a father's sperm chromatin accessibility do not lead to increase obesity or diabetes susceptibility in offspring. (A–B) Body weight gain (g) monitoring over 12 weeks on a Western diet: (A) females from both father types (F1_F0 CD_ + WD, n = 8; F1_F0__WD_ + WD, n = 15), (B) males (F1_F0 CD_ + WD, n = 17; F1_F0__WD_ + WD, n = 14). Statistical significance was assessed using two-way repeated measures ANOVA test. (C–D) Blood glucose level after 12 weeks of a Western diet: (C) females from both father types (F1_F0 CD_ + WD, n = 8; F1_F0__WD_ + WD, n = 15), (D) males (F1_F0 CD_ + WD, n = 17; F1_F0__WD_ + WD, n = 14). Statistical significance was assessed using Student's *t*-test. (E–F) Glucose tolerance test after 12 weeks of a Western diet: (E) females from both father types (F1_F0 CD_ + WD, n = 8; F1_F0__WD_ + WD, n = 15), (F) males (F1_F0 CD_ + WD, n = 17; F1_F0__WD_ + WD, n = 14). Statistical significance was assessed using two-way repeated measures ANOVA test. (G–H) Insulin tolerance test after 12 weeks of a Western diet: (G) females from both father types (F1_F0 CD_ + WD, n = 8; F1_F0__WD_ + WD, n = 15), (H) males (F1_F0 CD_ + WD, n = 17; F1_F0__WD_ + WD, n = 14). Statistical significance was assessed using two-way repeated measures ANOVA test. (I–J) Insulin level after 12 weeks of a Western diet: (I) females from both father types (F1_F0 CD_ + WD, n = 5; F1_F0__WD_ + WD, n = 7), (J) males (F1_F0 CD_ + WD, n = 8; F1_F0__WD_ + WD, n = 8). Statistical significance was assessed using Student's *t*-test. (K–L) Pyruvate tolerance test after 12 weeks of a Western diet: (I) females from both father types (F1_F0 CD_ + WD, n = 8; F1_F0__WD_ + WD, n = 15), (J) males (F1_F0 CD_ + WD, n = 17; F1_F0__WD_ + WD, n = 14). Statistical significance was assessed using two-way repeated measures ANOVA test.

Next, our objective was to ascertain if paternal obesity and the resulting alterations in sperm chromatin accessibility are linked to a greater likelihood for the offspring to acquire obesity or diabetes. To assess this, we provided a Western diet to 12-week-old F1 offspring of both control diet and Western diet fathers (F1_F0 CD_ and F1_F0__WD_) for 12 weeks. Interestingly, when adult F1 mice were subjected to a Western diet, both sexes saw an equivalent increase in body weight, regardless of the diet of their parents (Fig. 4A and B). We also could not detect differences in fasting glucose between these two groups (Fig. 4C and D). Considering the minor glucose homeostasis changes observed on the control diet (Fig. 2G and .3D), we next performed GTT, ITT, PTT, and measured fasting insulin concentration. These tests revealed no differences in glucose homeostasis between F1_F0__WD_ and F1_F0 CD_ mice fed with a Western diet (Fig. 4E-L). In summary, our findings indicate that paternal obesity does not make offspring more susceptible to an enhanced risk of developing obesity or diabetes, irrespective of gender. The detected alterations in sperm chromatin do not contribute to reshaping the metabolic features of the offspring. Nonetheless, it is possible that exposing offspring to an obesogenic environment early in life, during the perinatal stage, would cause the F1 offspring of a father fed a Western diet to have more prominent metabolic alterations. According to our data, the epigenetic memory of the paternal diet may be counteracted by a possible resilience or adaptive capacity that develops in the metabolism of the offspring. This phenomenon may be attributed to many compensating mechanisms present in the developing embryo that help reduce the likelihood of developing obesity or diabetes.

## 4 Conclusion

In summary, our research presents a nuanced view of the epigenetic influence of paternal obesity on offspring. Our data indicate that while paternal obesity does induce changes in sperm chromatin structure, these do not straightforwardly translate into metabolic disorders in the offspring. This indicates a form of interaction with genetic and environmental factors, protecting against the transmission of obesity and diabetes risks.

The minimal metabolic disturbances observed in our study emphasize the potential for biological systems to mitigate the impact of epigenetic modifications, suggesting a degree of adaptability in the face of inherited challenges. Our findings call for a deeper exploration of the underlying biological mechanisms and highlight the importance of considering natural mating conditions when assessing the impact of paternal epigenetics on subsequent generations.

## Funding

The study received funding by the Instituto de Salud Carlos III under the project PI20/00732 (co-funded by the European Regional Development Fund "A Way to Make Europe") (given to A.O). This work received supplementary funding from the projects PID2019-108175RB-I00 (financed by the MICIU/AEI/10.13039/501100011033) and 2021-SGR-01320 (from the 10.13039/501100002809Generalitat de Catalunya, Departament de Recerca i Universitats and CERCA Programme/Generalitat de Catalunya) (both given to M.C.). A.O. was awarded a Miguel Servet contract (CP19/00083) sponsored by the Instituto de Salud Carlos III and co-supported by the European Social Fund (FSE) under the "Investing in your future" initiative. I.T. is the recipient of the PRE2020-094896 fellowship, which is sponsored by the MCIN/AEI/10.13039/501100011033/and co-funded by the European Social Fund. S.R.L. is supported by the INVESTIGO program, grant 2022 INV-1 00028 (contract 100028TC3), which is funded by the European Union's "NextGeneration EU" and the 10.13039/501100003359Generalitat de Catalunya. R.H.T. is the recipient of a Ramón y Cajal contract (RYC2022-037070-I) supported by the MCIN/AEI/10.13039/501100011033/and co-sponsored by the European Social Fund (FSE+). M.T. is sponsored by a contract funded by the Instituto de Salud Carlos III using European money from the Recovery, Transformation, and Resilience Plan, under file code IHMC22/00039 and "Funded by the 10.13039/501100000780European Union - Next Generation EU."

## Data availability

The ATAC-Seq datasets generated and analyzed in this research can be found in the NCBI GEO repository with the accession number GSE263011.

## CRediT authorship contribution statement

**Iasim Tahiri:** Writing – review & editing, Visualization, Validation, Methodology, Investigation, Formal analysis. **Sergio R. Llana:** Writing – review & editing, Visualization, Formal analysis, Data curation. **Júlia Fos-Domènech:** Investigation, Formal analysis. **Maria Milà-Guash:** Investigation. **Miriam Toledo:** Investigation, Formal analysis. **Roberta Haddad-Tóvolli:** Investigation. **Marc Claret:** Writing – review & editing, Funding acquisition. **Arnaud Obri:** Writing – review & editing, Writing – original draft, Visualization, Validation, Supervision, Project administration, Methodology, Investigation, Funding acquisition, Formal analysis.

## Declaration of competing interest

The authors declare that they have no known competing financial interests or personal relationships that could have appeared to influence the work reported in this paper.
