# Supplementary material for: Paternal obesity induces changes in sperm chromatin accessibility and has a mild effect on offspring metabolic health
Source: Heliyon. 2024 Jul 5;10(14):e34043. doi: 10.1016/j.heliyon.2024.e34043 (PMC11296027; doi:10.1016/j.heliyon.2024.e34043)
Supplement: Multimedia component 1 [file mmc1.pdf]

Supplementary Figure 1

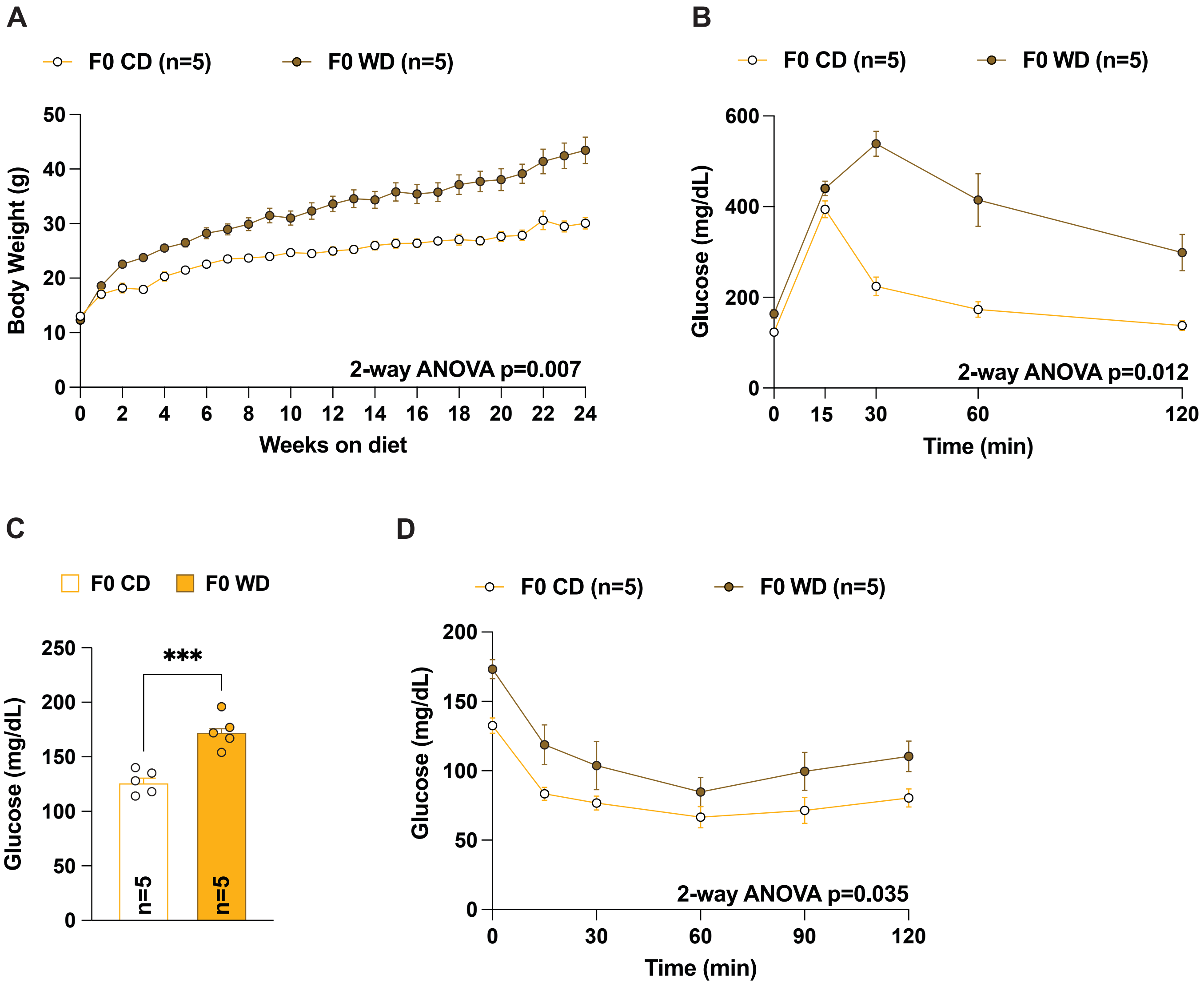

Supplementary Figure 2

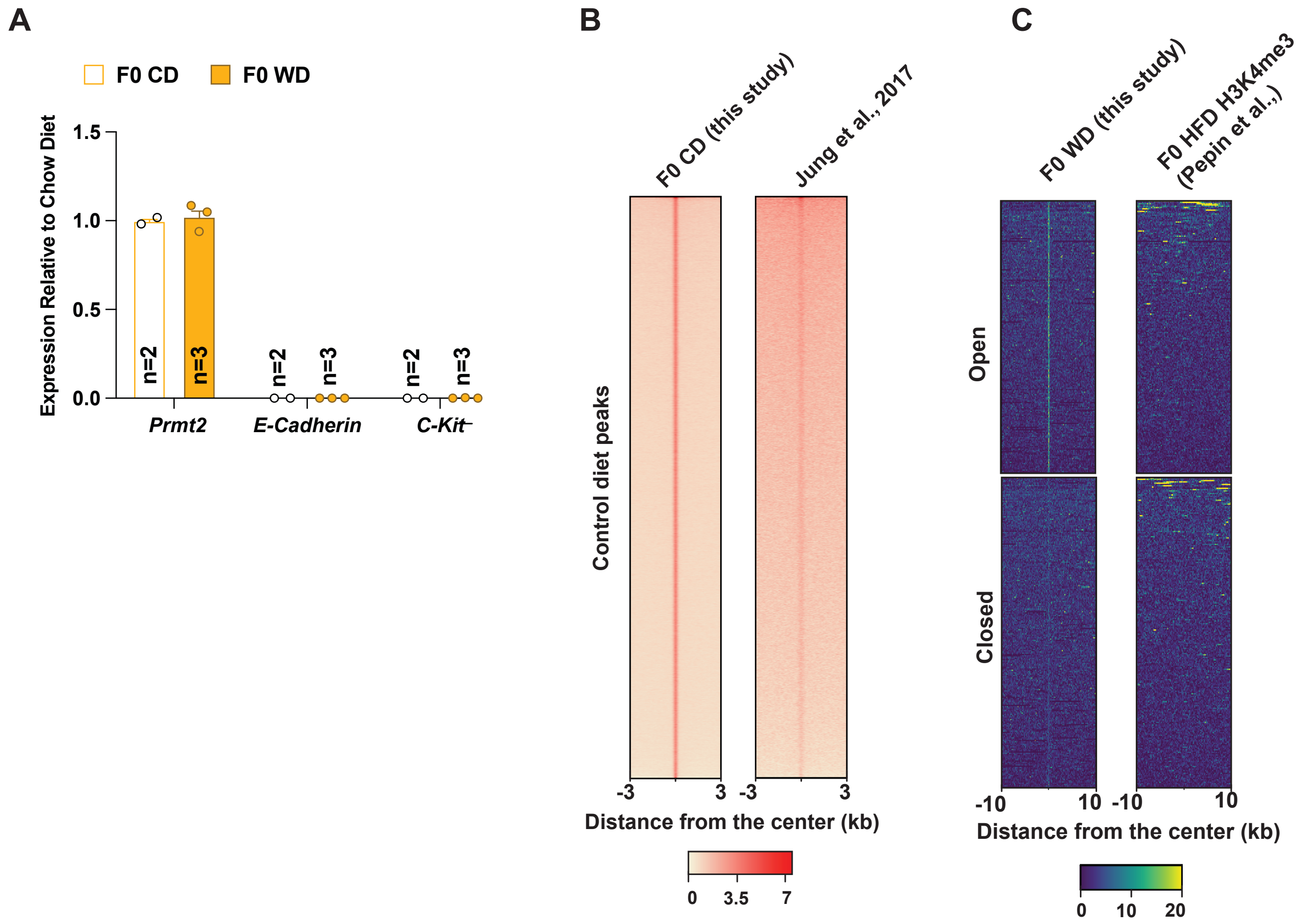

ATAC Differential accesibility analysis			Annotation	
**peaks**	**log2FoldChange**	**pvalue**	**SYMBOL**	**annotation**
chr6:42245772-42246010	1,973082	0,00321	Gstk1	Promoter (<=1kb)
chr7:126342254-126342495	1,885765	0,00195	Lat	Distal Intergenic
chr11:83860491-83860933	1,876331	0,00037	Hnf1b	Intron
chr15:71272840-71273152	1,830974	0,00597	Fam135b	Distal Intergenic
chr6:49317994-49318230	1,809829	0,00177	Ccdc126	Promoter (1-2kb)
chr2:81564596-81564815	1,787939	0,01463	Zfp804a	Distal Intergenic
chr5:43312767-43313099	1,707565	0,00667	Cpeb2	Distal Intergenic
chr8:11915975-11916227	1,662586	0,02384	Tex29	Distal Intergenic
chr12:57125484-57125698	1,654704	0,03193	Slc25a21	Intron
chr4:100473688-100473957	1,608047	0,00503	Ube2u	Distal Intergenic
chr12:91723272-91723506	1,575894	0,00561	Ston2	Intron
chr18:13136889-13137119	1,550129	0,01272	Hrh4	Distal Intergenic
chr10:38896310-38896571	1,549994	0,00261	Lama4	Distal Intergenic
chr1:102024931-102025238	1,515698	0,02104	Gm20268	Distal Intergenic
chr5:119508776-119509041	1,513465	0,00507	NA	Distal Intergenic
chr19:58774301-58774601	1,503019	0,01349	Pnliprp2	Intron
chr15:23833931-23834251	1,494503	0,02432	Cdh18	Distal Intergenic
chr5:79436876-79437167	1,493530	0,00051	Adgrl3	Distal Intergenic
chr8:121247669-121247994	1,487601	0,00089	Foxl1	Intron
chr3:8646338-8646618	1,486183	0,00399	Gm32496	Distal Intergenic
chr12:75805937-75806181	1,485214	0,00270	Syne2	Distal Intergenic
chr6:119615616-119615921	1,482961	0,02870	Wnt5b	Intron
chr4:58690157-58690486	1,482780	0,00331	Or2k2	Distal Intergenic
chr4:114294916-114295196	1,469139	0,00126	Trabd2b	Distal Intergenic
chr14:58207225-58207667	1,441942	0,00118	Fgf9	Distal Intergenic

Supplementary Figure 4

ATAC Differential accesibility analysis			Annotation	
**peaks**	**log2FoldChange**	**pvalue**	**SYMBOL**	**annotation**
chr2:38518589-38518858	-4,418181	0,000386	Nek6	Intron
chr7:67667198-67667441	-2,765586	0,000191	Ttc23	Promoter (<=1kb)
chr2:14981513-14981725	-2,505594	0,009388	Cacnb2	Promoter (<=1kb)
chr18:64408540-64408758	-2,442427	0,005345	Fech	Distal Intergenic
chr14:73543322-73543593	-2,435493	0,000517	Nudt15	Intron
chr6:95803151-95803560	-2,096273	0,007002	Suclg2	Distal Intergenic
chr19:17732772-17732999	-2,065646	0,015969	Pcsk5	Intron
chr14:120081870-120082160	-1,973665	0,010164	Oxgr1	Distal Intergenic
chr1:85527930-85528190	-1,960777	0,007218	Sp110	Intron
chr10:40550976-40551254	-1,945456	0,000320	Slc22a16	Distal Intergenic
chr7:88495942-88496193	-1,904985	0,015478	Rab38	Distal Intergenic
chr6:101939590-101939919	-1,875140	0,003960	Gm9871	Distal Intergenic
chr13:21292933-21293429	-1,868908	0,000555	Gpx5	Promoter (<=1kb)
chr13:53073542-53073743	-1,855409	0,011265	Nfil3	Distal Intergenic
chr6:148895579-148895798	-1,798927	0,019557	Caprin2	Promoter (<=1kb)
chr4:142494337-142494584	-1,785259	0,023380	Kazn	Distal Intergenic
chr9:27777120-27777431	-1,781247	0,000531	Opcml	Distal Intergenic
chr14:44740793-44741075	-1,774037	0,001456	Gm8267	Intron
chr18:33182329-33182613	-1,752654	0,000502	Stard4	Exon
chr16:30066549-30066799	-1,744906	0,014053	Hes1	Promoter (<=1kb)
chr8:92370268-92370600	-1,741641	0,027021	Irx5	Intron
chr1:184278115-184278370	-1,723009	0,015759	Dusp10	Distal Intergenic
chr14:8486851-8487131	-1,711152	0,001572	Fam3d	Intron
chr3:32597485-32597763	-1,701693	0,033589	Gnb4	Promoter (<=1kb)
chr14:18512868-18513141	-1,694744	0,008403	Ube2e2	Distal Intergenic

Supplementary Figure 5

A

Open				
**Motif**	**Name**	**q-value**	**Target %**	**Background %**
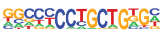	Zic3	$1x10^{-4}$	10.53	2.62
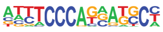	ZNF143	$4x10^{-3}$	6.70	1.93
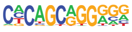	Zic2	$8x10^{-3}$	7.18	2.14
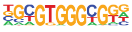	Egr2	$8x10^{-3}$	4.31	0.88
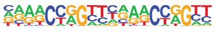	Tcfcp211	$8x10^{-3}$	4.31	0.9
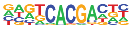	Npas4	$8x10^{-3}$	7.66	2.85
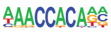	Runx1	$1x10^{-2}$	22.01	13.14
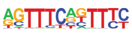	Irf3	$1x10^{-2}$	2.87	0.68
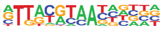	Nfil3	$1x10^{-2}$	7.18	2.88
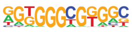	Klf14	$1x10^{-2}$	32.06	22
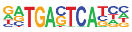	Jun-AP1	$1x10^{-2}$	4.78	1.68
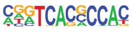	Srebp2	$1x10^{-2}$	4.78	1.38
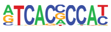	Srebp1a	$2x10^{-2}$	6.22	2.21

B

Closed				
**Motif**	**Name**	**q-value**	**Target %**	**Background %**
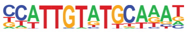	Oct4:Sox17	$1x10^{-3}$	3.8	0.9
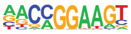	ETV1	$2x10^{-2}$	16.03	8.68
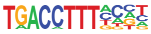	Nur77	$3x10^{-2}$	2.95	0.61
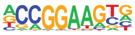	ETV4	$3x10^{-2}$	11.39	5.76
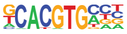	bHLHE40	$3x10^{-2}$	8.44	3.85
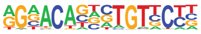	ARE	$3x10^{-2}$	5.91	2.33
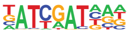	CUX1	$3x10^{-2}$	5.91	2.25
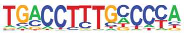	PPARE	$3x10^{-2}$	15.19	8.69
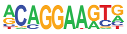	ERG	$3x10^{-2}$	19.41	12.17
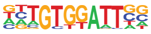	Foxh1	$3x10^{-2}$	10.97	5.69
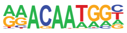	Sox15	$4x10^{-2}$	12.66	7.41
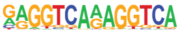	TR4	$4x10^{-2}$	2.53	0.33

Supplementary Figure 6

A

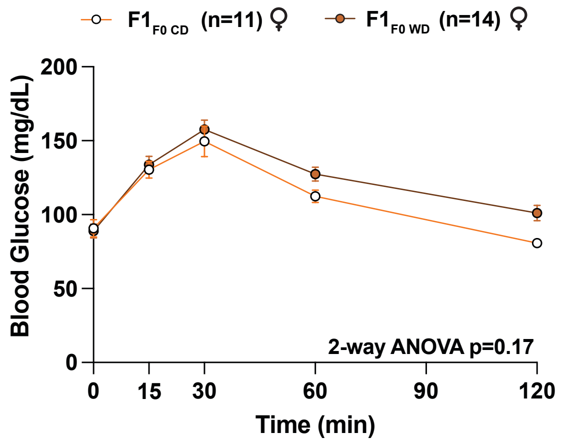

B

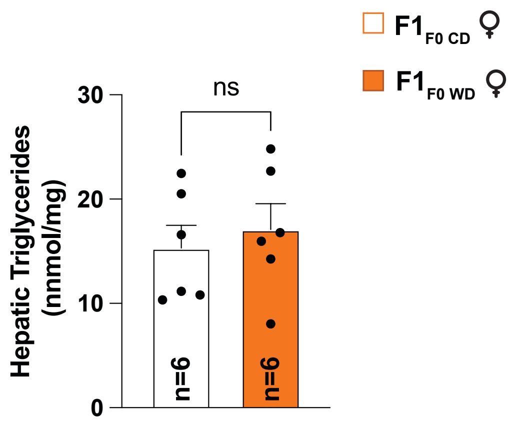

Supplementary Figure legends

Supplementary figure 1: Generation of obese and lean males.

(A) Body weight monitoring of male mice over six months on either a Western diet (F0 WD) or control diet (F0 CD). Statistical significance was assessed using two-way ANOVA test.

(B) Glucose tolerance test of male mice after six months on either a Western diet (F0 WD) or control diet (F0 CD). Statistical significance was assessed using two-way ANOVA test.

(C) Blood glucose level of male mice after six hours of fasting of F0 WD or F0 CD. Statistical significance was assessed using Student *t*-test.

(D) Insulin tolerance test of male mice after six months on either a Western diet (F0 WD) or control diet (F0 CD). Statistical significance was assessed using two-way ANOVA test.

Results are given as mean ± SEM. ***, P ≤ 0.001;

Supplementary figure 2: Quality control of mature sperm preparation.

(A) Expression levels of *Prmt1*, *E-Cadherin*, and *C-Kit* measured by quantitative PCR (qPCR) in mature sperm from mice on control diet (F0 CD, n=2) or Western diet (F0 WD, n=3).

(B) Heatmap depicting peak intensities with a color gradient, highlighting the similar pattern in sperm chromatin accessibility between our study and Jung et.al 2017.

C) Heatmaps of peak intensities of chromatin region sensitive to Western diet in sperm (open and closed) and of regions enriched with H3K4me3 in sperm (Pepin et al., 2022).

Supplementary figure 3: The top 25 open chromatin regions in sperm induced by a Western diet.

List of the open regions identified by ATAC-seq (top 25 region with a threshold for fold change (FC) of 1 and p-value < 0.05 are shown).

Supplementary figure 4: The top 25 closed chromatin regions in sperm induced by a Western diet.

List of the closed regions identified by ATAC-seq (top 25 region with a threshold for fold change (FC) of -1 and p-value < 0.05 are shown).

Supplementary figure 5: Transcription motif analysis in sperm chromatin open and closed regions.

Motif enrichment analysis conducted with HOMER for (A) open and (B) closed chromatin regions, indicating the top-scoring motifs, q-values (with a threshold for threshold q < 0.05), best-match transcription factors, and percentage of target versus background.

Supplementary figure 6: Glycerol tolerance test and hepatic triglycerides are not altered in females coming from obese fathers.

(A) Glycerol tolerance test at 12 weeks of age of females from both father types ($F1_{F0\ CD}$, n=11; $F1_{F0\ WD}$, n=14) Statistical significance was assessed using two-way repeated measures ANOVA test.

(B) Hepatic triglycerides levels after 6 hours of fasting at 12 weeks of age off females from both father types ($F1_{F0\ CD}$, n=6; $F1_{F0\ WD}$, n=6). Statistical significance was assessed using Student's *t*-test.
